# Technology-Based Self-Care Methods of Improving Antiretroviral Adherence: A Systematic Review

**DOI:** 10.1371/journal.pone.0027533

**Published:** 2011-11-30

**Authors:** Parya Saberi, Mallory O. Johnson

**Affiliations:** Department of Medicine, University of CaliforniaSan Francisco, San Francisco, California, United States of America; RAND Corporation, United States of America

## Abstract

**Background:**

As HIV infection has shifted to a chronic condition, self-care practices have emerged as an important topic for HIV-positive individuals in maintaining an optimal level of health. Self-care refers to activities that patients undertake to maintain and improve health, such as strategies to achieve and maintain high levels of antiretroviral adherence.

**Methodology/Principal Findings:**

Technology-based methods are increasingly used to enhance antiretroviral adherence; therefore, we systematically reviewed the literature to examine technology-based self-care methods that HIV-positive individuals utilize to improve adherence. Seven electronic databases were searched from 1/1/1980 through 12/31/2010. We included quantitative and qualitative studies. Among quantitative studies, the primary outcomes included ARV adherence, viral load, and CD4+ cell count and secondary outcomes consisted of quality of life, adverse effects, and feasibility/acceptability data. For qualitative/descriptive studies, interview themes, reports of use, and perceptions of use were summarized. Thirty-six publications were included (24 quantitative and 12 qualitative/descriptive). Studies with exclusive utilization of medication reminder devices demonstrated less evidence of enhancing adherence in comparison to multi-component methods.

**Conclusions/Significance:**

This systematic review offers support for self-care technology-based approaches that may result in improved antiretroviral adherence. There was a clear pattern of results that favored individually-tailored, multi-function technologies, which allowed for periodic communication with health care providers rather than sole reliance on electronic reminder devices.

## Introduction

As HIV infection has evolved from an acute to a chronic illness, much of the medical treatment of HIV-positive patients has shifted from critical care to outpatient settings. Consequently, self-care practices of individuals living with HIV have emerged as a significant topic for disease treatment and management [1], [2], [3], [4], [5], [6]. Optimal adherence to antiretroviral (ARV) therapy is among the most important aspects of these practices and an emergent strategy to improve ARV adherence is the use of technology-based methods. The strength of technology lies in its ability to transcend borders, cultures, and languages; therefore, understanding self-care technology-based strategies used by HIV-positive individuals to improve adherence is critical for providers and researchers who seek to support patients in enhancing adherence while simultaneously utilizing existing resources and limiting cost.

Individual self-care has been defined in numerous ways [7], [8], [9], [10], [11], [12]. A broad definition of self-care refers to “those activities individuals undertake in promoting their own health, preventing their own disease, limiting their own illness, and restoring their own health [7], [8], [9].” These activities are generally informed by technical knowledge of health care professionals and lay experience, but are undertaken without professional support. Self-care has also been defined as the “naturalistic decision making process involving the choice of behaviors that maintain physiologic stability (maintenance) and the response to symptoms when they occur (management)” [11]. Therefore, self-care maintenance includes health-promoting habits, adhering to treatment regimens, and monitoring and managing symptoms. More explicitly, HIV-specific self-care behaviors include ARV adherence and engagement in care [13].

High ARV adherence is associated with enhanced CD4+ cell count, reductions in HIV viral load, and decreased morbidity and mortality [14], [15], [16]. Conversely, non-adherence may result in virologic rebound, ARV drug resistance, transmission of drug-resistant virus, and progression to AIDS [17], [18], [19], [20], [21]. Despite the necessity of high adherence, in the U.S. and Europe the percentage of prescribed doses taken has been estimated to range from 60–70% [22], [23], [24], [25], [26], [27]. “Forgetfulness” is commonly cited as the top reason for missing doses [28]; therefore, many researchers have investigated the role of electronic reminder devices, such as alarms and pagers, to improve adherence. The U.S. Department of Health and Human Services [29], the British HIV Association [30] and the World Health Organization [31] have acknowledged the supportive role of technology-based methods to improve adherence. This recognition underscores the need for stronger evidence of the effectiveness of these technologies and the identification of cost-containing strategies for improving adherence.

We conducted a systematic review of studies that explored the use and impact of technology-based methods by HIV-positive individuals for improving ARV adherence. The purpose of this review was to extend prior reviews examining the impact of electronic reminder devices [32] and the efficacy of interventions [33] on adherence. Specifically, we focused on the use of self-care technology-based adherence strategies.

## Methods

### Objective

The primary objective of this systematic review was to evaluate the impact of self-care technology-based methods on ARV adherence. We report the efficacy (adherence, HIV viral load, and CD4+ cell count) and other secondary outcomes of using self-care technology-based methods.

### Data Sources

Initially, we searched PubMed, EMBASE, Cochrane Central, Web of Science, and PsycINFO from 1/1/1980 through 12/31/2010. Additionally, we screened the references of all pertinent articles to identify additional relevant publications.

### Search Strategy

The search strategy was in the style of Cochrane Highly Sensitive Search Strategy [34] for identifying reports of randomized, non-randomized, observational, and qualitative studies in PubMed, as well as the appropriate MeSH terms, and a wide range of relevant search terms in all databases. The detailed search strategy used for PubMed can be found in Table S1 This strategy was modified as appropriate for use in other databases. We included all quantitative and qualitative studies (including descriptive studies) published in the English language.

### Inclusion/Exclusion Criteria

We included research regarding the impact of technology-based methods used by HIV-positive individuals on our primary outcomes (ARV adherence, HIV viral load, and CD4+ cell count), secondary outcomes (quality of life, adverse effects, and feasibility/acceptability data), and outcomes of qualitative/descriptive studies (interview themes, reports of use, and perceptions of use). Among quantitative studies with our primary outcomes, findings were contrasted across groups receiving and not receiving the intervention or in before-after comparisons. Only studies published in English but regardless of geographical location were included in the review.

Technology-based methods were defined as devices such as electronic reminder devices (including alarms, electronic pillboxes, and pagers), mobile telephones (for automated functions such as automated text messages and automated alarms), personal digital assistants (PDAs), computer software, and Internet and mobile applications. These included tools that may have been initially set up or implemented by a researcher/clinician, but that the participant/patient could use independent of the researcher/clinician for adherence self-care. This decision was made to set apart self-care techniques that an individual could utilize independent of their health-care providers from methods that required constant interaction/supervision of a health professional. Therefore, reviewed studies did not include adherence monitoring devices (e.g., medication event monitoring systems or MEMs) or any method that clinicians used to monitor patients' adherence to give feedback. We did not include studies that examined technologies that facilitated the interactions between patients/participants and clinicians/researchers (such as email, text messaging, or telephone) because we did not view these methods as strictly promoting self-care. Multifactorial interventions containing at least one self-care technology-based method were included.

### Review Method and Data Abstraction

Using the EndNote software package, relevant studies were located in the above-mentioned data sources and duplicates and irrelevant articles were removed by one author. One author and the research assistant read the remaining citations and identified eligible studies based on pre-specified inclusion/exclusion criteria. All uncertainties and disagreements were arbitrated by the second author. Using a data abstraction form, one author and the research assistant summarized pertinent information from included articles.

### Outcome Variables

Primary outcomes included ARV adherence (based on self-report, pill-counts, pharmacy refill records, MEMS), HIV viral load, and CD4+ cell count. Secondary outcomes consisted of quality of life, adverse effects, and feasibility/acceptability data. For qualitative and descriptive studies, interview themes, reports of use, and perceptions of use were summarized.

## Results

**Figure 1:**
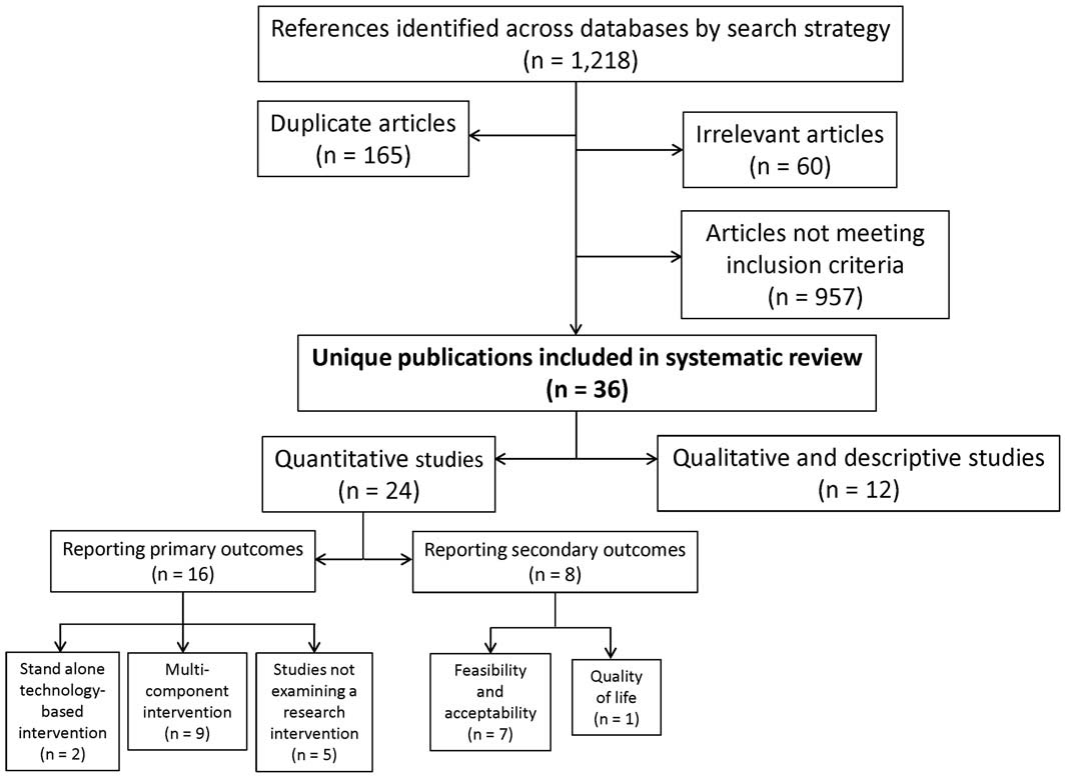
Selection process for study inclusion.

From 1,207 gross results, 36 publications met our eligibility criteria and were included (Figure 1). Among these publications, 24 were quantitative, from which 16 reported on our primary outcomes (adherence, viral load, CD4+ cell count) [35], [36], [37], [38], [39], [40], [41], [42], [43], [44], [45], [46], [47], [48], [49], [50] and 8 stated information regarding our secondary outcomes (quality of life and feasibility/acceptability) [51], [52], [53], [54], [55], [56], [57], [58]. Table 1 and Table S2 summarize these studies. An additional 12 qualitative and descriptive studies were identified that are summarized in Table S3 [59], [60], [61], [62], [63], [64], [65], [66], [67], [68], [69], [70].

**Table 1:** Summary of quantitative studies with primary outcomes.

Source	Country, City/StateStart-End YearSample sizeAge (years)% Male% MSM% BL% WH	Inclusion / Exclusion Criteria	Study Objectives	If examined interventions, description of intervention	Length of follow-up	Outcomes			
						Method of Adherence Assessment: IntervalAdherence Outcomes	Viral load (copies/mL)	CD4+ cell count (cells/mm^3^)	Other Outcomes
Andrade et al, 2005 [35]Wu et al, 2006 [58]	United States, Maryland1999–2000N = 58Mean age = 3859%N/R88%N/R	Inclusion: ≥18 years, able to self-medicate, care at Johns Hopkins Moore HIV Clinic, treatment-naïve & initiating ARV or treatment-experienced & switching ARV (had ≤3 prior ARV regimens)Exclusion: inability to self-medicate, severe dementia, institutionalization	Assess if use of Disease Management Assistance System (DMAS: programmable medication reminder device providing verbal reminders at ARV dosing times) device improves ARV adherence, viral load, and CD4+.	Control: Monthly individualized 30-minute adherence counseling session+standardized adherence feedback transcript (education on barriers of adherence & hazards of non-adherence)Intervention: Same as controls+DMAS	24 weeks	- EDM & self-report: 4 days- Overall mean adherence by electronic drug-exposure monitoring caps: 80% in DMAS vs 65% in control (NS)	- Undetectable viral load: 34% in DMAS vs 38% in controls (p = 0.49)- 1log10 reduction: 72% in DMAS vs 41% in controls (p = 0.02)- Mean reduction: −2.1log10 DMAS vs −0.98log10 controls (p = 0.02)	Mean CD4+: 301+/−172 DMAS vs 250+/−172 controls (p = 0.28)	Post-hoc analysis:- Mean adherence in memory impaired (n = 31): 77% DMAS vs 57% controls (p = 0.001)- Mean adherence in memory intact: 83% DMAS vs 77% controls (p = 0.25)QOL: controls had improved & DMAS had reduced QOL score.
Fairley et al, 2003 [36]	Australia, Melbourne2001–2002N = 43Mean age = 3898%91%N/RN/R	Inclusion: ≥18 years, not planning to interrupt or change ARVs in next 3 month, had missed at least 1 dose of treatment by self-report in last month	Determine if a comprehensive adherence package improved self-reported ARV adherence pre- and post-intervention.	Use of adherence package: an educational program (on HIV, HIV treatment, importance of adherence), medication planner, & choice of adherence aids (pillbox, text messaging at scheduled doses, or medication alarm).	5 months	- Self-report: 4, 7, 28 days- Missed doses decreased in last 4 days (0.76 to 0.38, p = 0.03) & last 7 days (1.5 to 0.74, p = 0.005), but not last 28 days (2.5 to 2.5, p = 0.63)- Morisky score: pre- intervention = 2.9, post- intervention = 3.3 (p = 0.006)	Undetectable viral load: 73% pre-intervention vs 74% post-intervention (p = 1.0)	Mean CD4+: 513 pre-intervention vs 551 post-intervention (p = 0.8)	Report of use: 17 began timed pillbox, 13 used plain pillbox, 11 SMS text, 6 declined aids
Golin et al, 2002 [37]	United States, North Carolina1998–1999N = 117Mean age = 3880%N/R26%16%	Inclusion: English/ Spanish speaking, newly initiating PIs or NNRTIs	Examine relationship between adherence & patient factors, regimen factors (adherence aids such as medication lists, timers, & pillboxes), clinical interaction, & social factors.	None	48 weeks	- MEMS, pill count, self-report: 4 weeks- Adherence in those using no adherence aids = 67.5% vs adherence = 76% among top quartile of adherence aid users (p = 0.01)			
Iroha et al, 2010 [38]	Nigeria, Lagos2008N = 21243%: 60–119months48.1%N/RN/RN/R	Inclusion: Caregivers of children who had been on ARVs for at least 30 days & who consented to participate	Determine level of ARV adherence among pediatric patients & barriers & facilitators of adherence according to caregivers.	None	None	- Self-report: 3 days- Use of reminders not associated with adherence- 30% of those adherent used reminders vs 31% of those non-adherent			
Kalichman et al, 2005 [39]	United States, GeorgiaN/RN = 446Mean age = 40.684%56%77%19%	Inclusion: Use of internet at least once monthly in past 3 months	Examine Internet use in HIV+ adults, including use of Internet for health, social support, & non-health/social support. Examine characteristics of those who use Internet for health-related information.	None	None	- Self-report: 7 days- 1.9 times odds (95% CI = 1.2–3.2) of missing medications in those not using Internet for health information vs those who did use Internet for health information	Odds of undetectable viral load for those who did not use Internet for health was 0.9 times (95% CI = 0.6–1.6) vs those who did use Internet for health	- CD4+ not associated with Internet use (adjusting for active coping & education)- CD4+ related to Internet use (adjusting for education)	
Kalichman et al, 2001 [40]	United States, WisconsinN/RN = 112Median age = 380%0%88%9%	N/R	Compare information, motivation, behavioral skills, & use of specific ARV adherence strategies in HIV+ women who had missed ≥1 dose in past week to women who were adherent to ARVs in past week.	None	None	- Self-report: 7 days- Those who missed a dose more likely to have ever used pillboxes & datebooks- A trend in those who missed doses for greater past use of timers & beepers- No difference between groups for current use of strategies			Report of use: 33 of those who missed & 39 of those who adhered reported using adherence strategies such as timers, beepers, pill boxes, reminder notes, & date books
Levy et al, 2004 [41]	Australia, Melbourne2002–2002N = 68Mean age = 4287%68%N/RN/R	Inclusion: ≥18 years,, obtained ARVs from the AlfredExclusion: Those planning on interrupting or changing treatment within next 3 months or those reporting 100% adherence with undetectable viral load	Determine the impact of education-based adherence intervention on adherence.	Adherence aids (pillboxes, electronic alarms) plus general HIV education plus individualized ARV counseling (given computerized medication planner) plus availability of pharmacist pager for urgent advice or adherence problems.	20 weeks	- Self-report: 4, 7, 28 days- Decrease in missed doses: in last 4 days decrease from 1.9 to 1; in last 7 days decrease from 3 to 1.8, in last 28 days decrease from 7.4 to 4.2 (all p<0.001)- Improved Morisky score (1.3 to 0.5, p = 0.001)	Viral load: pre-intervention = 21,801, post-intervention = 17,264 (p = 0.39)	- CD4+: pre-intervention = 382, post-intervention = 406 (p = 0.70)- CD4%: pre = 20%, post = 19.5% (p = 0.83)	
Lyon et al, 2003 [42]	United States, Washington1998–2000N = 23Age range = 15–2334.8%N/R100%0%	N/R	Develop a pilot program to increase ARV adherence among HIV+ youth & involve families & peers in this effort (where youth asked to identify adult family member or adult friend who could act as their treatment buddy).	Biweekly group meetings to discuss topics (e.g., purpose of ARV therapy, managing AEs, provider communication, etc), +education session for youth & family separately, +joined interactive review using game show format. On alternative weeks, only youth met to discuss medications & adherence devices (pillboxes, beepers, calendars, wrist watches with alarms).	12 weeks	- Self-report: 2 weeks- Increased ARV adherence between study start and end- Miss ≥1 dose yesterday: start = 50%, end = 12%- Miss ≥1 dose in past 2 days: start = 43%, end = 18%- Miss ≥1 dose in past 2 weeks: start = 78%, end = 36%- “forgot” as reason for missing: study start = 43%, study end = 40% (alarm watch did not seem effective even though rated as best of the 5 adherence aids)	4 youths had viral load reduction to undetectable during group	At 6 months: 4 youth had improved CD4+ to >500	Report of use: In qualitative interviews, caregivers thought that some interventions that would help youth with adherence would be: 1) videotapes featuring teens with HIV, 2) vibrating beepers that hold pills, 3) watches with alarm.
Mannheimer et al, 2006 [43]	United States, 24 states1999–2003N = 928Mean age = 3878%N/R55%25%	Inclusion: ARV-naïveExclusion: sites already using interventions similar to the study for most patients	Assess efficacy of medication managers (MM) or alarms (ALR) in ARV-naïve HIV+ persons with virologic failure occurring on or after 4-month follow-up visit.	2×2 factorial-Intervention:1) MM: individualized, structured, long-term adherence support from MM using IMB model2) ALR: individually programmed alarm3) MM+ALRControl: standard of care	30 days (median)	- Self-report: 3 days- MM vs no-MM: higher rate of reporting 100% adherence (OR = 1.42, p<0.001)- ALR vs no-ALR: no significant difference for adherence	- MM vs no-MM: 13% lower rate of 1^st^ virologic failure on or after 4 months (p = 0.13)- ALR vs no-ALR: rate of 1^st^ virologic failure was 25% *higher* in ALR (p = 0.02)	- MM vs no-MM: higher mean increase in CD4+ from baseline (22.5 higher in MM, p = 0.01)- ALR vs no-ALR: no difference	QOL:MM vs no-MM: no significant differenceALR vs no-ALR: no significant difference
Murphy et al, 2002 [44]	United States, CaliforniaN/RN = 79Mean age = 3988%N/R46%30%	Inclusion: ≥18 yrs, prescribed ARVs, English speaking, not participating in other medication adherence study or other clinical trial, no psychiatric conditions making patient unable to participate in group experience, having difficulty with medication adherence (missed doses once/week or more)	Test hypothesis that patients assigned to multidisciplinary & multicomponent intervention condition (using behavioral strategies, simplified patient information, & social support) are more likely to be adherent to ARVs than those in standard of care condition.	Intervention: 5 group (information on HIV treatment & adherence, modify/strengthen adherence plan, etc) & 2 individual sessions (identify barriers & adherence plan, gain control over health care, & communication with medical provider). Behavioral strategies consisted of pillboxes, wrist alarms, & beepers.Control: standard of care	3 months	- Self-report: 3 days & 1 month- From immediate post-intervention to 3-month follow-up, a trend for intervention group to not taking doses any later than 1 hour of scheduled time vs controls (p = 0.06)- No difference in self reported adherence- Decline in use of behavioral strategies from baseline to 3 months in control group (p = 0.01)			
Murphy et al, 2007 [45]	United States, CaliforniaN/RN = 141Mean age = 39.982.4%N/R47.9%23.2%	Inclusion: ≥18 yrs, prescribed ARVs, ARV non-adherent (miss ≥1×/wk), English-speaking, receiving care at AIDS Healthcare Foundation, CD4+>100, no opportunistic infections 1 month prior to enrollment, not participating in other medication adherence or clinical trials, no psychiatric condition (schizophrenia, bipolar)	Test hypothesis that patients assigned to intervention condition (behavior change strategies, social support, & simplified patient education information) would be more likely to be adherent than those in standard of care.	Intervention: 5 sessions using behavioral strategies (simple reminder strategies, self-monitoring, medication preparation systems, etc) & cognitive-behavioral techniques (communication skills, etc), simplified HIV information, social support. 4 booster sessions to review intervention & patient experience, a jeopardy-like adherence game, & review of adherence barriers & problem-solving.Control: standard care	9 months	- Self-report, pill count, MEMS: various intervals3 months: no difference for any adherence measure9 months: intervention MEMS adherence = 70% & pill-count = 78%; control MEMS adherence = 59% & pill-count = 69%From 3 to 9 months:- Intervention: increase % dose adherence (p = 0.05), marginal effect for % days adherence (p = 0.06), no change pill-count adherence- Control: no change dose adherence, decline % days adherence (p = 0.02), decline pill-count adherence (p<0.01)			
Safren et al, 2003 [46]	United States, MassachusettsN/RN = 70N/R80%67%30%N/R	Inclusion: Adherence <90% at 2 weeks & return for 2week assessment	Test feasibility, utility, & efficacy of customizable pager, programmed using web-based technology, to increase & maintain adherence in those with pre-existing adherence problems.	After 2 weeks of monitoring adherence, those with <90% adherence randomized:Intervention: receive a pagerControl: continue monitoring	12 weeks	- MEMS: 2 & 12 weeks- Pager group had more adherence improvement vs controls (P<0.004)- Pager group: baseline 55% adherence, 70% at week 2 & 64% at week 12; control arm, had 57% adherence at baseline, 56% in week 2, & 52% at week 12			
Samet et al, 2005 [47]	United States, Massachusetts1997–2000N = 151Mean age = 42.981%23.5%47%30%	Inclusion: current or lifetime history of alcohol problems (2 or more positive responses to CAGE screening questionnaire or clinical diagnosis), on ARV, English or Spanish fluency, Mini-Mental State Exam score ≥21, no plans to move from Boston in 2 years	Assess effectiveness of an individualized multi-component intervention (including watch with timer) to promote ARV adherence in a cohort of HIV+ individuals with history of alcohol problems.	Intervention: 4 encounters with RN to address alcohol problems, provide watch with programmable timer, enhance perception of treatment efficacy, & deliver individually tailored assistance to facilitate medication use (exploring ways to tailor medications)Control: standard of care	13 months	- Self-report: 3 & 30 days- No statistically significant difference in adherence between intervention & control groups from baseline to 6 months or baseline to 12 months	No significant difference in viral load	- No significant difference in CD4+	Subgroup analysis: No significant difference in primary or secondary outcomes in subgroups (gender, hazardous drinking, adherence ≥95%, IDU in past 6 months, viral load <500, CD4+ ≤350)
Samet et al, 1992 [48]	United States, Massachusetts1990N = 83Median age = 3680%28%20%46%	Inclusion: ≥18 yrs, current ZDV use	Determine extent of & clinical variables (including timers) associated with ZDV adherence.	None	None	- Self-report: 1 & 7 days- Variable associated with >80% ZDV adherence was use of medication timer (OR = 4.4, 95% CI = 1.0–19.1)- Most common reasons for missing were “forgot…” (75%) & “did not have the medication with me” (43%)			
Simoni et al, 2010 [49]	China, Beijing2006–2008N = 70Mean age = 3681%N/RN/RN/R	Inclusion: Mandarin-speaking at Ditan Hospital, ≥18 yrs, CD4<350, eligible for ARV, willing to & physically capable of attending follow-up visits at the hospital.Exclusion: Cognitively impaired & actively psychotic	To evaluate the feasibility & initial efficacy of a nurse-delivered adherence intervention among HIV+ outpatients initiating ARVs in Beijing, China.	All received 1 educational session, daily medication schedule, pillbox, & referral to peer support group, then randomized:Intervention: choice of electronic reminder device (their cell phone or study reminder device), 3 counseling sessions alone or with adherence partner (formulating daily medication schedule, setting reminder strategies, etc), or both reminder & counseling.Control: standard of care	25 weeks	- Self-report & EDM: 7 & 30 daysSelf-report: 100% adherence more likely at 13, 19, 25weeks in intervention armEDM: Intervention arm had greater dose & on-time adherence than control (NS)Longitudinal analysis: >2-fold increased odds of 100% adherence for intervention vs control for cumulative effect of average weekly improvements between 7 & 13 weeks (OR = 2.23, 95% CI = 1.05–4.72, p = 0.04)	- Both arm showed comparable improvement in viral load over time (NS).- No difference between arms in longitudinal analysis.	- No statistically significant difference between arms in CD4+ gain.- No difference between arms in longitudinal analysis.	Feasibility:- Minimal attrition.- Of 28 who opted for counseling, 75% completed 2 or 3 sessions.- 12 opted for counseling with treatment adherence partner (mainly spouse or partner).- 26 opted for alarm: 7 used own cell phone & remaining used study alarm without problem.
Simoni et al, 2009 [50]	United States, Washington2003–2007N = 224Mean age = 4076%N/R30%47%	Inclusion: ≥18 yrs, proficient in English, living within service area of the pager, initiating or changing at least 2 ARVsExclusion: Cognitively impaired, actively psychotic, or known history of harming others	Determine relative efficacy of peer support & pager messaging strategies in improving med adherence & clinical outcomes among those initiating or switching to a new ARV regimen.	All met with pharmacist, nutritionist, case manager, then randomized:Intervention:1) Peer support: 6 twice monthly gatherings & weekly phone calls.2) Pager messaging: customized pager; 3 pages daily for 2 months, then tapered in last month; confirmation return page requested; messages included dose reminders; educational; adherence assessment; entertainment3) Both peer & pagerControl: standard of care	9 months	- Self-report & EDM: 7 daysPeer vs no peer:- A 2-fold increased odds of 100% adherence between 2 weeks & 3 months (95% CI = 1.1–4.01, p = 0.02)- Finding did not persist after 6 & 9 monthsPager vs no pager:- Did not predict improved odds of 100% adherence at 3 or 9 months, but was marginally associated with *decrease* in 100% adherence at 6 months (OR = 0.5, 95% CI = 0.24, 1.03, p = 0.06)	- No peer effect for viral load- No pager effect at any time point for viral load	- No peer effects for CD4+- No pager effect at any point for CD4+	Post-hoc analysis:- Peer meetings did not predict adherence differences, but greater attendance associated with reduced viral load at 3, 6 & 9 months- Attendance marginally associated with CD4+ at 3months- More pager response predicted significant reduction in log10 viral load at 3 & 9 months- Pager response predicted significant increase in CD4+ at 3, 6, & 9 months

ARV: antiretroviral; BL: Black/African-American; CI: confidence interval; DMAS: Disease Management Assistance System device; EDM: electronic drug monitoring; IMB model: Information, Motivation, Behavioral Skills model; IDU: injection drug use; MEMS: Medication Event Monitoring System (electronic drug monitoring); MSM: men who have sex with men; MM: medication managers; N/R: not reported; NS: not statistically significant; OR: odds ratio; QOL: quality of life; RN: registered nurse; SMS: short message service; vs: versus; WH: White; ZDV: Zidovudine.

### Quantitative Research

#### Publications with Primary Outcome

These 16 studies were mainly published between 2001 through 2010 (with the exception of one published in 1992 [48]) and were primarily conducted in the U.S. (75%). Baseline sample size ranged from 23–928 (median = 98); from studies where mean age is presented, mean age ranged from 36–43 years; percentage of male participants ranged from 0–98% (median = 80%); and within the U.S. studies, the percentage of participants who were Black ranged from 20–100% (median = 47%). The most common method of adherence assessment was self-report (63%), followed by a combination of self-report and another method (such as MEMS caps and pill counts) (31%), and solely MEMS caps (6%).

*Studies not examining a research intervention-* In five of the 16 studies, the relationship between technology-based methods and adherence was reported [37], [38], [39], [40], [48]. These studies presented conflicting results on the positive [37], [39], [48] or neutral [38], [40] effect of these strategies on adherence. In the only quantitative study examining Internet use among regular Internet users [39], those who did not use the Internet to seek health information were more non-adherent than those who used it for this purpose.

*Stand-alone technology-based interventions-*Two studies reported the effect of stand-alone technology-based interventions [35], [46], consisting of electronic reminder devices, such as a pager or a programmable medication reminder device providing verbal reminders. In participants receiving individualized adherence counseling sessions, the use of the Disease Management Assistance System (DMAS) device, an electronic device that produces a timed voice message to prompt subjects to take ARVs, resulted in a mean adherence of 80% in the intervention arm versus 65% in the control arm, which was not statistically significant [35]. Post-hoc analyses noted an effect in memory impaired individuals. Surprisingly, the use of DMAS was associated with some deterioration in quality of life (see “Publications with Secondary Outcomes”) [58]. Safren and colleagues reported a statistically significant increase in adherence with the use of pagers; however, this improvement was not clinically significant, as adherence remained poor (≤70%) at points of outcome assessment in both arms of the study [46].

*Multi-component interventions including a technology-based method-*We identified nine publications that examined the effects of multi-component interventions, including a self-care technology-based method [36], [41], [42], [43], [44], [45], [47], [49], [50]. These interventions also included individualized counseling appointments [36], [41], [43], [47], [49], [50], group sessions [42], [45], or a combination of one-on-one and group sessions [44]. The technology-based adherence strategies consisted of mobile telephone automated text messages, alarms, beepers, and wrist watches with alarms. In these studies, four reported enhanced ARV adherence [36], [41], [42], [49], two revealed a trend for statistically significant improvements [44], [45], two showed mixed results (improved adherence with counseling support but not with electronic reminder devices) [43], [50], and one did not result in changes in adherence [47]. Increased CD4+ cell count was observed in one publication [42]; however, the remaining studies either did not detect any changes [36], [41], [43], [47], [49], [50] or did not report this value [44], [45]. In one study, an increase in the number of individuals with undetectable viral load was reported [42], five did not detect any changes in viral load [36], [41], [47], [49], [50], two did not report this outcome [44], [45], and one showed a statistically significant increased rate of virologic failure with the use of reminder devices [43]. The median length of follow-up in these studies was 20 weeks (range = 4–52 weeks).

In a 2-by-2 factorial design study, including a medication manager or medication alarm, the use of individualized, structured, long-term adherence support strategies from trained medication managers was associated with higher reports of perfect adherence and 13% lower rates of virologic failure in comparison to no medication manager [43]. However, use of a medication alarm did not produce a significant difference in adherence but resulted in 25% higher rates of virologic failure in comparison to not using medication alarms. Similarly, participants were randomized in another 2-by-2 factorial design to a peer-support intervention or a pager messaging strategy [50]. The use of pager did not result in increased odds of reporting 100% adherence; however at six months, there was a trend for decreased adherence.

### Publications with Secondary Outcomes

These eight studies were published between 2000 and 2010 and 75% were conducted in the U.S. Sample sizes ranged from 10–300 (median = 30); mean age ranged from 31–43 years; and percentage of male participants ranged from 0–88% (median = 56%).

*Feasibility and acceptability*- From the seven publications that evaluated feasibility and acceptability of technology-based self-care methods [51], [52], [53], [54], [55], [56], [57], all concluded that these methods were feasible and acceptable. Among these studies, four examined the use of a single technology-based method, which included mobile telephones [51], automated pagers [52], [55], smaller timers [55], pillboxes with timer [55], PDAs [56], and patient-education video [57]. In using a pager as a technology-based method to improve adherence [52], most individuals expressed interest in its use for medication reminders and entertaining messages (news bulletins, jokes, and quizzes). However, the foremost reported disadvantage of the pager was its size. In one study, despite participants indicating that remembering to take ARVs as problematic, the use of reminder interventions alone did not result in improvements in adherence at two months [55].

Two studies assessed feasibility and acceptability of multi-component interventions that included technology-based self-care methods (e.g., alarms, beepers, and alarms watches), as well as non-technology-based methods, such as integration of medications into daily life, use of pillboxes, etc [53], [54]. The Client Adherence Profiling-intervention Tailoring protocol included diagnosis of the adherence problem and selection of interventions based on patient factors, treatment regimen, and the patient-provider relationship [53]. In another study [54], the intervention consisted of multiple sessions with a nurse practitioner trained in motivational interviewing. In both studies, methods to improve adherence were discussed with the participants and their application and utilization were monitored. A high proportion of participants reported using reminders in these studies.

*Quality of life-* Wu and colleagues conducted a secondary data analysis to assess the impact of DMAS on quality of life (Table 1) [58]. As described previously, DMAS is a medication reminder tool that transmits verbal messages at ARV dosing times [35]. At six months, individuals in the control arm had improved quality of life scores, whereas those in the intervention arm had deterioration in this score. Plausible explanations were that the use of DMAS could have been a negative reminder of the patient's HIV status or that due to its size and loud sound, DMAS may have threatened confidentiality.

### Qualitative and Descriptive Research

Twelve qualitative and descriptive studies were found in which technology-based methods were mentioned by the participant or the study specifically assessed the use of technology in improving adherence (Table S3) [59], [60], [61], [62], [63], [64], [65], [66], [67], [68], [69], [70]. These methods included mobile telephone alarms and reminders, beepers, watches, pagers, and other reminder devices. Studies were published between 2000 and 2010 and 67% were conducted in countries outside of the U.S [59], [60], [61], [63], [67], [68], [69], [70]. Sample sizes ranged from 12–330 (median = 43); in studies reporting mean age, mean age ranged from 36–50 years; and percentage of male participants ranged from 35–100% (median = 75%).

The self-reported use of technology-based methods (mainly electronic reminder devices) ranged from 3–23%. The reports of use of pillboxes, medication schedules, incorporation of medications into daily schedule, and friend/family support for adherence were common themes that emerged in various studies. Several studies reported that participants used more than one adherence tool [67], [68], [70].

In one study assessing the perception toward use of mobile telephones and PDAs [60], participants reported willingness to use these methods. However, in addition to the reminder function of these strategies, most wanted the ability to obtain information on HIV and communicate with providers. Similarly, in a study of the use of mobile telephone interventions, many participants requested to receive additional information on advancements in HIV medicine and research and to increase their communication with clinic providers [69]. Most did not view these reminders as intrusive; however, the majority preferred receiving only 1–2 reminders per week. Lastly, in one study, participants evaluated the pager system positively but the overall response rate was low and decreased dramatically over time [62]. The authors speculated that maintaining participants' interest over time, tapering nonessential messages, allowing user opt-out of certain features, and addressing device problems may result in a higher response rate.

## Discussion

In this systematic review, we evaluated the utilization of self-care technology-based methods by HIV-positive individuals to improve ARV adherence. Despite the fact that “forgetfulness” is commonly cited as a cause of non-adherence [28], the use of technology-based methods that solely remind patients to take ARVs at dosing times do not seem to be the most effective methods of enhancing adherence. As noted in qualitative studies, only a small proportion of individuals reported the use of reminder devices or found these methods helpful. The exclusive use of these electronic reminder devices has been shown to lead to slight improvements in ARV adherence [46], deterioration in quality of life [58], and a paradoxical effect on HIV viral load [43]. These devices may be useful in those who are memory-impaired [35] or in those whose “forgetfulness” is actually due to not remembering. The explanation for this seemingly contradictory evidence may lie in investigating the underlying cause of the reported “forgetfulness”, such as stigma, depression, drug and alcohol use, lack of social support, etc.

Results of both qualitative and quantitative studies indicate that participants are interested in using technology-based methods, but are most receptive toward the provision of a combination of reminders along with information regarding HIV treatment and enhanced communication with providers. In fact, quantitative intervention studies that include a fusion of individualized counseling sessions with a provider or a peer, as well as the *choice* of an adherence aid seemed to produce the most beneficial effects on adherence [36], [41], [42], [49]. This need for additional support was most evident in two-by-two factorial design studies where the efforts of medication managers and peers resulted in higher reporting of 100% adherence; however, the use of medication reminder devices did not produce this effect [43], [50]. In two studies, the combined use of education and technology-based methods did not enhance ARV adherence [35], [47]. We believe that the reason for this neutral result may be that one study [35], may not have had enough power to detect a statistically significant difference. In the second study [47], the study population consisted of individuals with alcohol problems; therefore, this risk factor may have impeded adherence and needed to have been addressed more thoroughly.

In order to provide context to the results of this review, we included qualitative studies where participants provided narratives of using technology-based methods. Furthermore, we included pilot and multi-component studies that incorporated the use of technology-based strategies. Therefore, the results of our study should be viewed in light of methodological differences across studies. Many studies examined interventions with multiple components; therefore, we cannot tease apart the independent effect of technology-base methods for improving adherence. Additionally, many studies relied on patient self-report to assess adherence which tends to over-estimate the actual level of adherence and is prone to the problem of recall bias. Lastly, we cannot rule out publication bias in that studies with negative results are less likely to be published.

Based on this review, it seems that the optimal characteristics of adherence-enhancing interventions that include a self-care technology-based method may involve: 1) tools that are easy to use, familiar to the patient, and that do not attract much attention (such as a personal mobile telephone) [49], [52], [53], [55], [58], [59], [60], [61]; 2) individually-tailored methods that are customized based on the patient's specific reasons for ARV non-adherence (such as the choice of technology-based methods) [36], [41], [43], [44], [49], [62], [65]; 3) multiple components, including the periodic involvement of providers and peers that provide education and support [36], [41], [42], [44], [45], [49], [50], [54], [69]; 4) multi-function strategies that include components to increase information (e.g., HIV treatment knowledge and consequences of non-adherence), motivation (e.g., treatment benefits and concerns), and behavioral skills (e.g., methods of enhancing adherence) [36], [41], [42], [43], [44], [45], [49], [52], [54], [60], [69].

Currently, there are several ongoing projects listed in Clinicaltrials.gov or the NIH Research Portfolio Online Reporting Tools (RePORT) that examine the effect of self-care technology-based methods of improving ARV adherence [71], [72], [73], [74], [75], [76], [77], [78], [79], [80]. The tools utilized focus on mobile telephones, such as use of automated text messaging and reminders [71], [72], [74], [75], [78], [79]; computer-delivered programs [73], [76]; and Web-based applications [77], [80]. The computer interventions include programs designed to promote health literacy in a tailored and interactive manner [73] and electronic versions of an intervention entitled Life Step [76]. Web-based interventions consist of online peer support programs [77] and behavioral health modules [80]. Therefore, it is apparent that much of the forth-coming studies have taken a tailored approach to the use of technology to enhance information, motivation, and behavioral skills. However, more research incorporating the above-mentioned characteristics of adherence-enhancing self-care technology-based interventions is needed to examine rules for adapting the technology to the individual and the optimal amount of each intervention component.

In 2008, an estimated $13.7 billion was spent on HIV programs [81]; however, less than half of those requiring HIV treatment are receiving ARVs [82]. Therefore, as we move toward the goal of universal access for HIV therapy [83], the consideration for careful budgeting and comprehensive utilization of existing resources is exceedingly important. Individually-tailored multi-component interventions including self-care technology-based methods may empower HIV-positive individuals, aid over-burdened clinics, and have the potential to result in cost-containment, while improving ARV adherence. Future research should focus on standardizing these interventions and testing the efficacy of simple, individually-tailored, multi-function technologies, which allow for the periodic involvement of health care providers.

## Supporting Information

Table S1 **Example of search strategy used in PubMed.**
(XLSX)

Table S2 **Summary of quantitative studies with secondary outcomes.**
(XLSX)

Table S3 **Summary of quantitative studies with secondary outcomes.**
(XLSX)
